# Supplementary material for: Declining grassland canopy height in China under asymmetric biomass allocation
Source: Nat Commun. 2026 Mar 3;17:3364. doi: 10.1038/s41467-026-70275-9 (PMC13066366; doi:10.1038/s41467-026-70275-9)
Supplement: Supplementary file 1 — Supplementary Information [file 41467_2026_70275_MOESM1_ESM.pdf]

Supplementary Information

Declining grassland canopy height in China under asymmetric biomass allocation

Huaqiang Li[1,2,†], Xinmiao Hu[1,†], Fei Li[1,3,4,*], Yingjun Zhang[3,*], Kejian Lin[1],

Jie Wang[3], Jiating Wang[5]

[1]Institute of Grassland Research, Chinese Academy of Agricultural Sciences, Hohhot, IM, China.

[2]Tasmanian Institute of Agriculture, University of Tasmania, Launceston, Tasmania, Australia.

[3]College of Grassland Science and Technology, China Agricultural University, Beijing, China.

[4]Key Laboratory of Grassland and Agricultural Ecological Remote Sensing, Ministry of Agriculture and Rural Affairs, Hohhot, IM, China.

[5]National Livestock Husbandry Station, Beijing, China.

[†]These authors contributed equally: Huaqiang Li and Xinmiao Hu.

*Corresponding authors.

Fei Li (lifei01@caas.cn) and Yingjun Zhang (zhangyj@cau.edu.cn).

This PDF file includes:

Supplementary Note 1 to Note 3

Supplementary Fig. 1 to Fig. 9

Supplementary Table 1 to Table 3

Supplementary Note 1: Additional experiment on fractional vegetation cover calculation

As a comparison, the grassland fractional vegetation cover was calculated using the classical gap fraction method, which is primarily based on the leaf area index (LAI) (Equation S1). The maximum LAI composites during the peak growing season (July–August) from 2001 to 2022 were computed using the Google Earth Engine (GEE) platform. To more accurately estimate the fractional vegetation cover of each grassland type, an iterative optimization was employed to select the *G* function of Equation S1, as detailed in Table S2. With these *G* functions, the grassland fractional vegetation cover model obtained an improved accuracy (Fig. S2b). Thereafter, we generated gridded fractional vegetation cover for Chinese grasslands from 2001 to 2022 using the fractional vegetation cover models (Equation S2). The results indicated that fractional vegetation cover calculated from the classical gap fraction method exhibited a significant increasing trend, with a rate of 0.23% y^{-1} (Fig. S4a). Spatially, 72% of Chinese grasslands showed an upward trend over the past 22 years (Fig. S4b). At the pixel scale, about 66% of the grassland aboveground biomass and fractional vegetation cover trends were significantly positively correlated (Fig. S4c). Additionally, we observed that fractional vegetation cover changes strongly explain aboveground biomass changes, especially in Inner Mongolia (Fig. S4d). Overall, the results from the classical gap fraction method were consistent with those obtained using the optimized linear spectral mixture analysis (SMA) method. The formula for the classical gap fraction method is as follows:

$$P_0(\theta) = e^{-\lambda_0 \frac{G(\theta,\theta_1)}{\cos\theta} \cdot LAI} \quad (S1)$$

$$\text{Fractional vegetation cover} = 1 - P_0(0) \quad (S2)$$

where $P_0(\theta)$ represents the gap fraction, where θ denotes the observation direction. θ_1 corresponds to the average leaf angle (ALA), and $G(\theta, \theta_1)$ refers to the orthogonal projection of a unit leaf area in the direction of θ. The parameter λ_0 indicates leaf dispersion and is set to 1, which reflects a random distribution of foliage. LAI is a measure of total leaf area per unit ground area and is derived from MOD15A2H. Based on the definition of fractional vegetation cover, the calculation is implemented with θ set to 0.

Supplementary Note 2: Additional experiment on canopy height trending robustness

To further assess the canopy height trending robustness, grassland canopy height in each grassland type was estimated using a linear model on the average division of surveyed canopy height by binning the [aboveground biomass/fractional vegetation cover] ratio (Fig. S5b). The results show that grassland canopy height across China exhibited a consistently declining trend in spatial patterns and the overall changing rate (Fig. S6). This suggests that the selection of percentile division for canopy height unit conversion does not affect grassland canopy height trending robustness.

Supplementary Note 3: Uncertainty analysis of canopy height estimation

From 2001 to 2022, we applied an [aboveground biomass/fractional vegetation cover] ratio strategy to map the spatial patterns and temporal changes of grassland canopy height, showing high accuracy (Fig. S5). However, optical remote sensing images may generate modeling errors along the fractional vegetation cover gradient, particularly in low-cover areas due to the influence of soil signals. Accordingly, we evaluated modeling uncertainties in estimating aboveground biomass, fractional vegetation cover, and canopy height across different grassland types, using R^2 and relative error, given the pronounced fractional vegetation cover gradient among Chinese grassland types, which ranges from 28% in alpine desert-steppe to 88% in temperate montane meadow. The results indicated that aboveground biomass simulations integrating climatic variables with satellite-derived metrics achieved the highest performance, whereas models relying solely on satellite metrics exhibited larger biases (Fig. S9a). In addition, the lowest fractional vegetation cover modeling performance was observed in the sparsely vegetated alpine desert-steppe, but application of the classical gap fraction method reduced these biases, thereby improving both R^2 and relative error (Fig. S9b). The above results indicated that modelling uncertainties in fractional vegetation cover were most pronounced in sparsely vegetated areas and may propagate into canopy height estimation, with a lower performance observed in alpine desert-steppe (Fig. S9c). Nevertheless, alpine desert-steppe constitutes only 5.2% of Chinese grasslands, occurring primarily on the western

Tibetan Plateau, and therefore its influence is confined to the magnitude of canopy height change rates rather than the overall declining trend. Overall, we conclude that, although optical remote sensing entails unavoidable uncertainties in sparsely vegetated regions, the relative temporal changes remain robust.

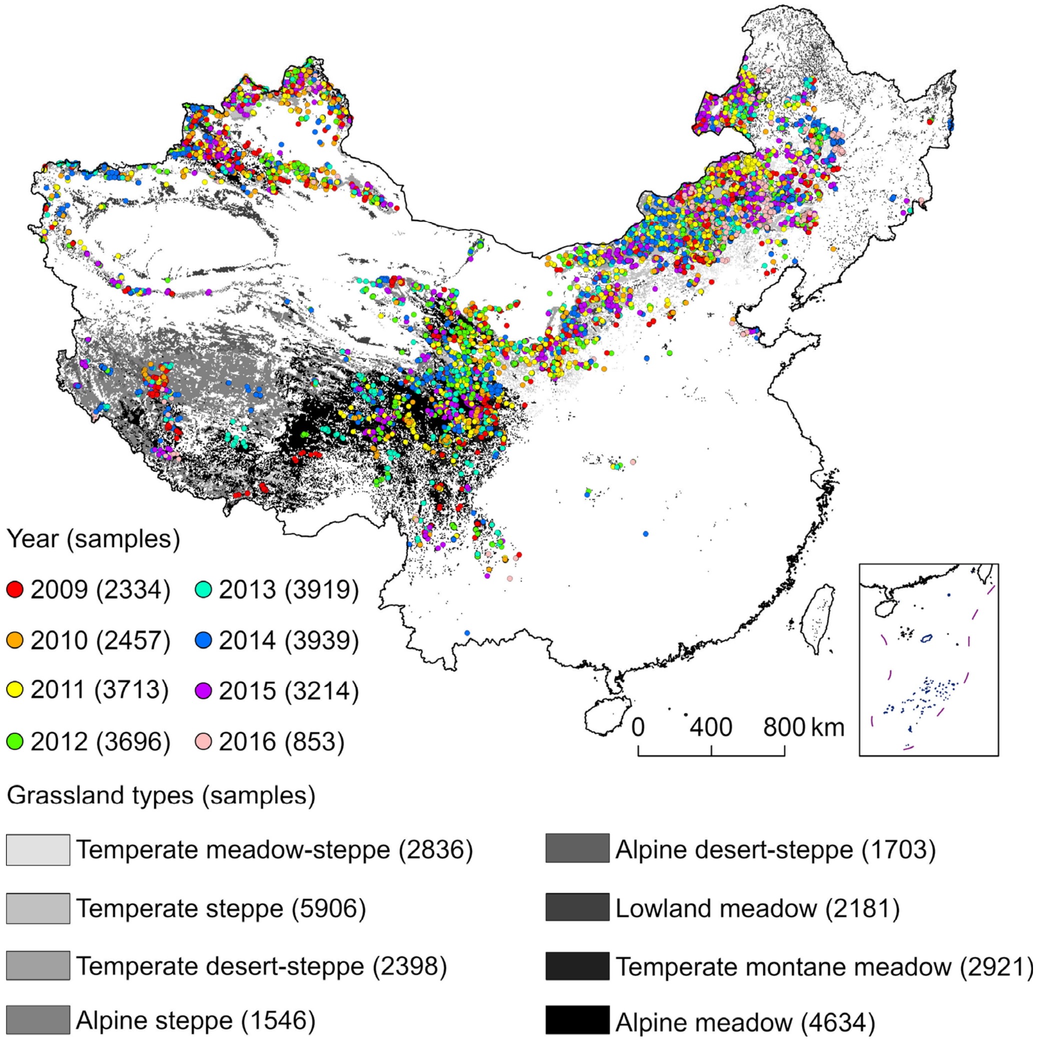

Supplementary Fig. 1. Spatiotemporal distribution of ground-truth samples over Chinese grasslands. Numbers inside the parentheses indicate the number of samples for each year and different grassland types.

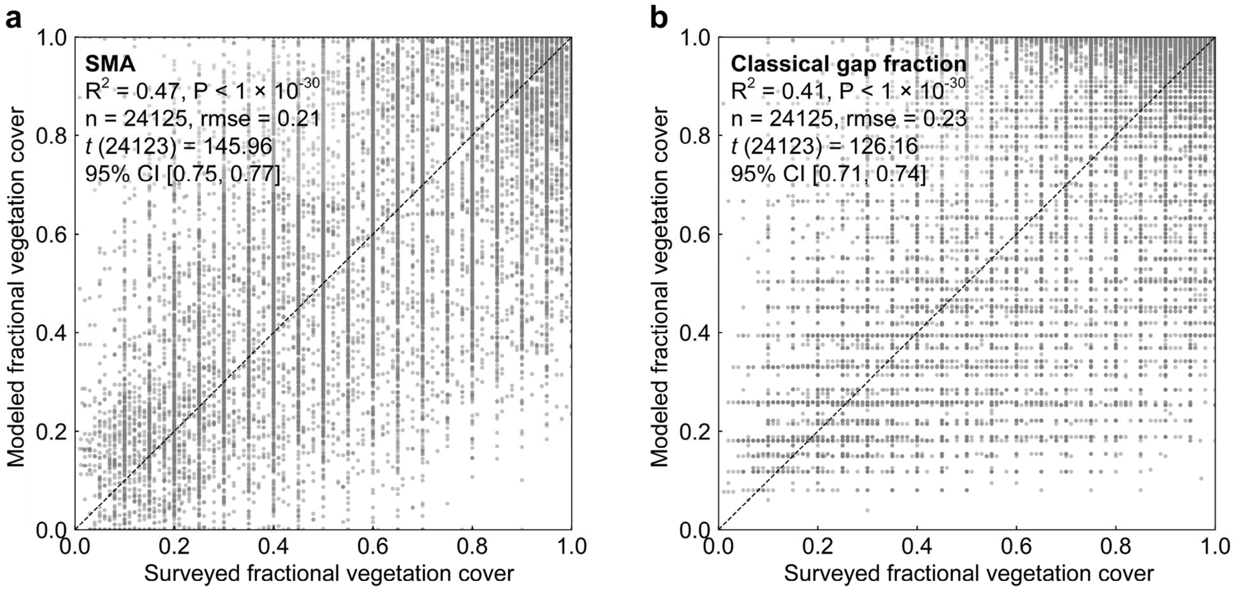

Supplementary Fig. 2. Performance of fractional vegetation cover modeling using SMA (a) and classical gap fraction (b). All the statistical significance in this study is assessed using a two-sided t-test, and no adjustment is made for multiple comparisons.

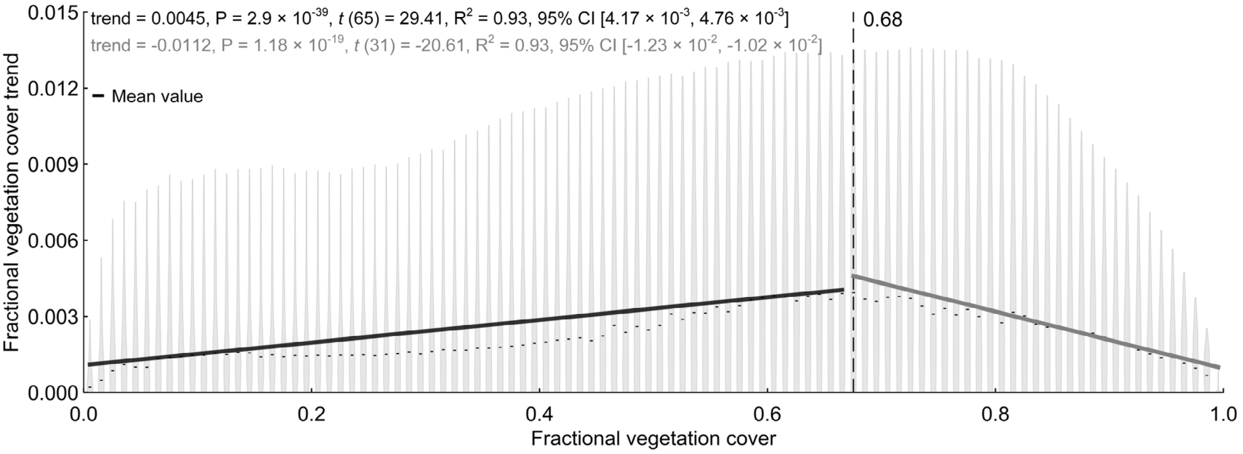

Supplementary Fig. 3. Fractional vegetation cover trending strength along the order of fractional vegetation cover from 0 to 1, where the violin plots show the kernel density distribution and mean value of fractional vegetation cover trends by binning fractional vegetation cover with an interval of 0.01.

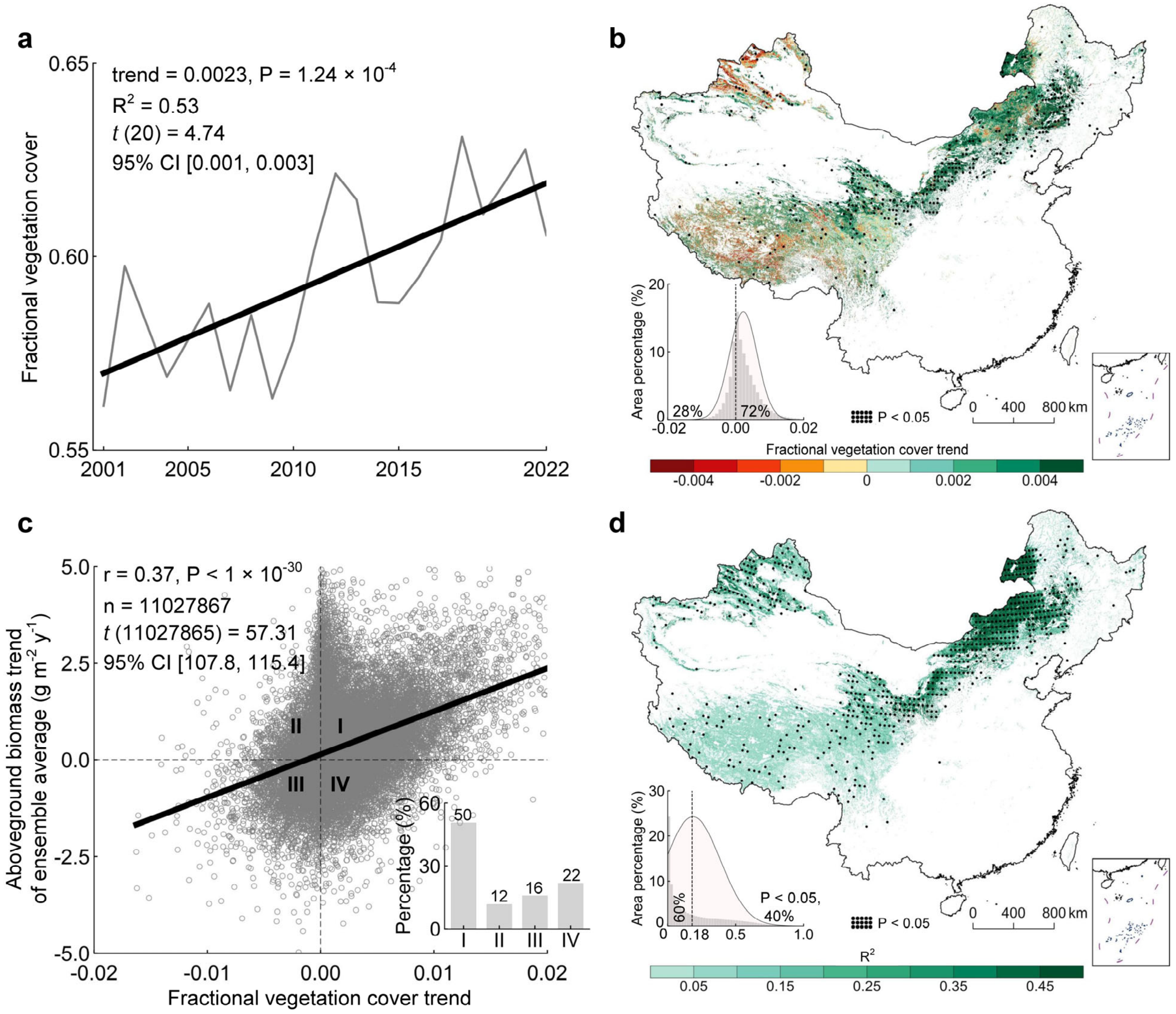

Supplementary Fig. 4. Changes in fractional vegetation cover derived from the classical gap fraction method and the correlation with aboveground biomass across Chinese grasslands from 2001 to 2022. a, Interannual changes in fractional vegetation cover. b, Spatial regime in fractional vegetation cover trends. c, Correlation between changing rates in fractional vegetation cover and aboveground biomass on a pixel basis. d, Spatial patterns of R^2 between changes in fractional vegetation cover and aboveground biomass; R^2 are dotted if statistically significant ($P < 0.05$). The embedded histograms of b, c, and d are statistics of area percentage aggregated by binning pixel values, with an overlaid normal curve for visualisation.

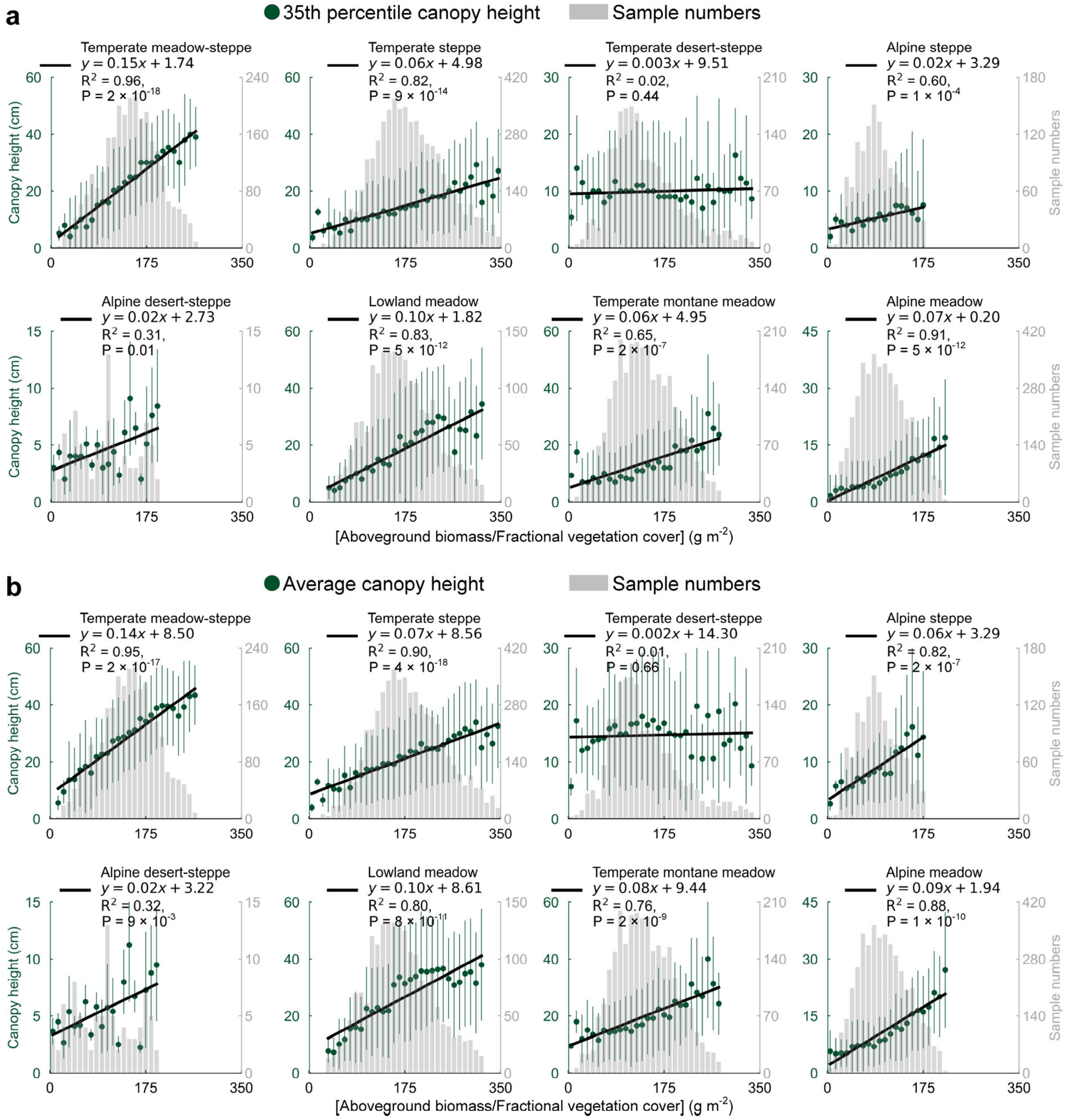

Supplementary Fig. 5. Relationships between [aboveground biomass/fractional vegetation cover] ratio and surveyed canopy height at different percentile divisions. a, 35th percentile division. b, Average division. The gray bars underneath represent the number of ground-truth samples used for statistics. The canopy height is presented as 35th percentile ± SD (a) and mean ± SD (b).

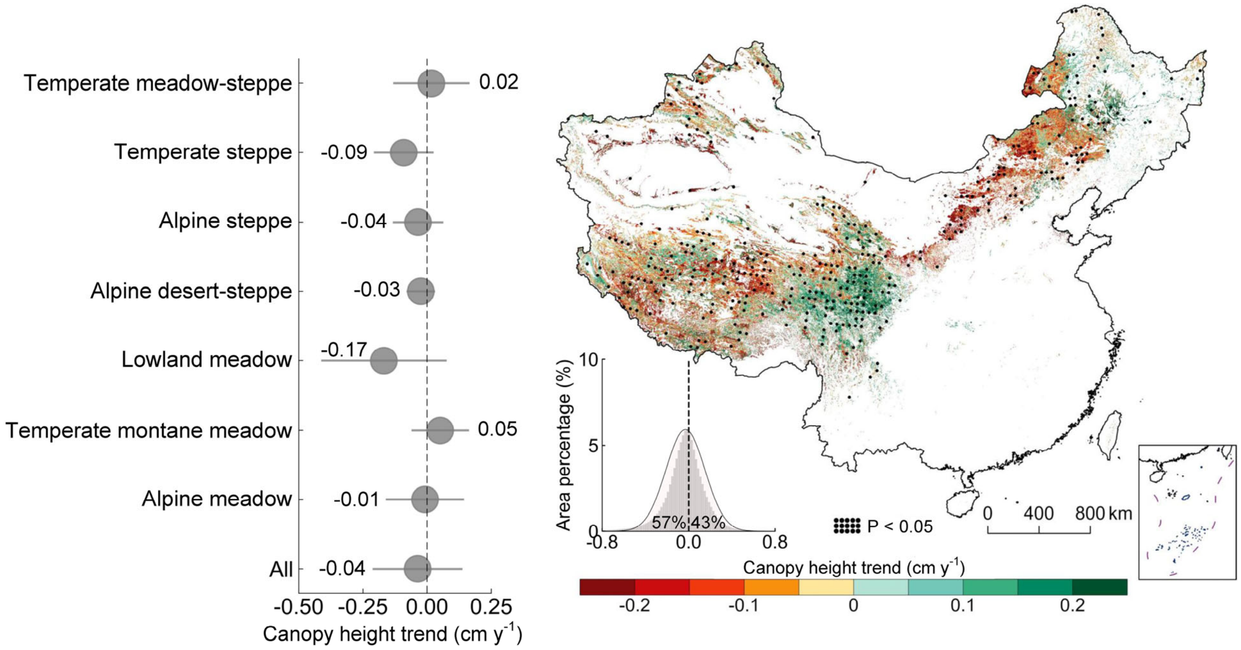

Supplementary Fig. 6. Trend rates of grassland canopy height for seven grassland types (left panel) and trend map (right panel), with dotted trends indicating statistical significance ($P < 0.05$). In particular, grassland CHs are converted from [aboveground biomass/fractional vegetation cover] ratios based on the average division of surveyed canopy height. The canopy height trends in the left panel are presented as mean ± SD. The embedded histograms of the right panels are statistics of area percentage aggregated by binning pixel values, with an overlaid normal curve for visualisation.

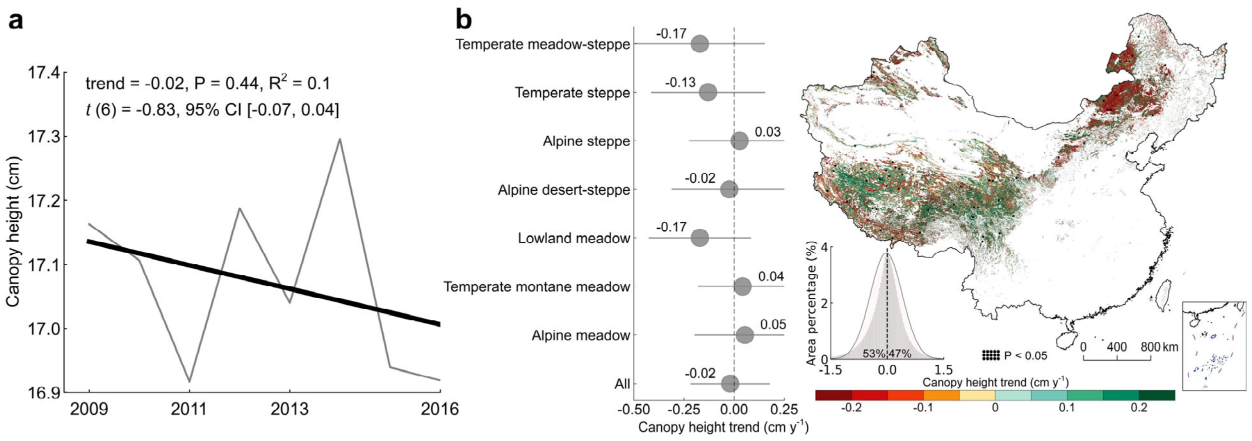

Supplementary Fig. 7. Spatiotemporal changes in grassland canopy height in China from 2009 to 2016. a, Interannual changes. b, Rates of grassland canopy height change for seven grassland types (left panel) and the trend map (right panel), where the trend is dotted if statistically significant ($P < 0.05$); the canopy height trends in the left panel are presented as mean ± SD; the embedded histograms in the right panels are statistics for the area percentage aggregated by binning pixel values, with an overlaid normal curve for visualisation.

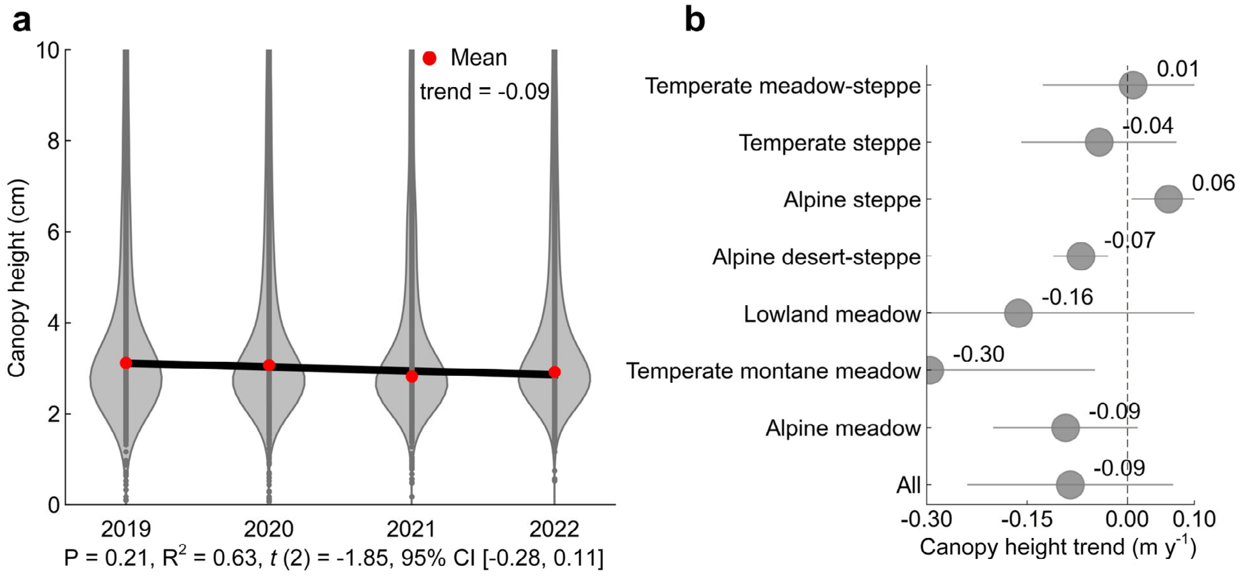

Supplementary Fig. 8. Changes of canopy height derived from the Global Ecosystem Dynamics Investigation (GEDI) dataset across Chinese grasslands from 2019 to 2022. a, Interannual changes of canopy height; violin plots show the kernel density distribution of canopy height for each year. b, Rates of canopy height change for seven grassland types; the data are presented as mean ± SD.

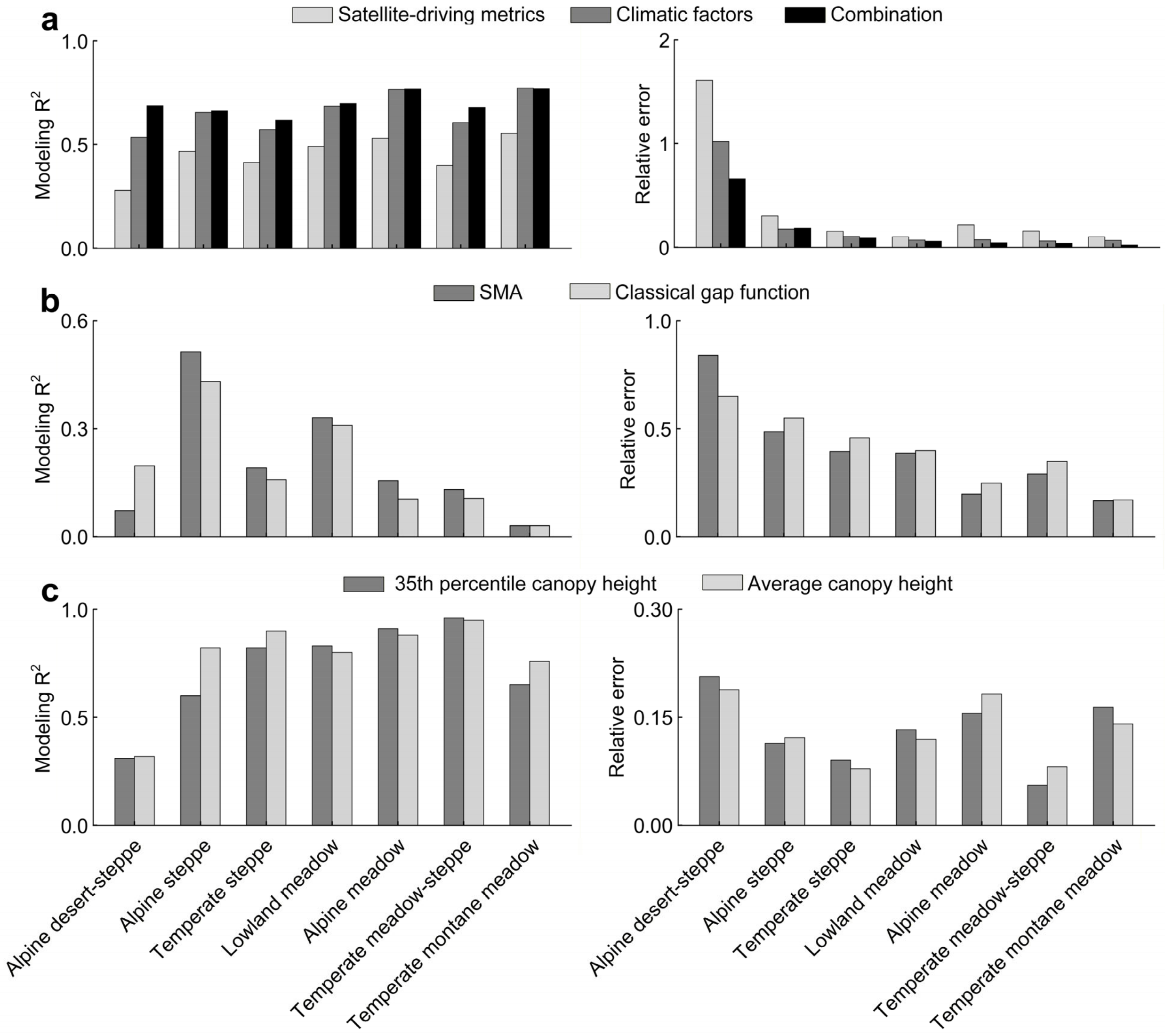

Supplementary Fig. 9. Modeling uncertainties across different grassland types ranked by fractional vegetation cover gradients, ranging from 28% in alpine desert-steppe to 88% in temperate montane meadow. The left panel shows the histogram of R^2, and the right panel presents the corresponding relative errors. a, Aboveground biomass. b, Fractional vegetation cover. c, Canopy height. SMA refers to the optimized linear spectral mixture analysis method.

Supplementary Table 1. Summary of driving variables for aboveground biomass modeling.

**Category (resolution)**	**Variable (unit)**	**Definition**
Satellite-driving metrics (500 m)	Red	Red band
	NIR	Near-infrared band
	Blue	Blue band
	Green	Green band
	SWIR1	Shortwave Infrared at 1240 nm
	SWIR2	Shortwave Infrared at 1640 nm
	SWIR3	Shortwave Infrared at 2130 nm
	NDVI	Normalized difference vegetation index
	EVI	Enhanced vegetation index
	EVI2	2-band enhanced vegetation index
	NIR_V	Near-infrared reflectance of vegetation
Climatic factors (~10km)	MAP (mm)	Mean annual precipitation from 2001 to 2022
	MAT (°C)	Mean annual temperature from 2001 to 2022
	MAV (kPa)	Mean annual vapor pressure deficit from 2001 to 2022
	MAR (MJ m^{-2})	Mean annual surface net solar radiation from 2001 to 2022
	P_{r_anom} (mm)	Annual precipitation anomaly from 2001 to 2022
	T_{a_anom} (°C)	Annual temperature anomaly from 2001 to 2022
	VPD_{anom} (kPa)	Annual vapor pressure deficit anomaly from 2001 to 2022
	R_{a_anom} (MJ m^{-2})	Annual surface net solar radiation anomaly from 2001 to 2022
	$P_{r\_anom\_gs}$ (mm)	Growing season precipitation anomaly from 2001 to 2022
	$T_{a\_anom\_gs}$ (°C)	Growing season temperature anomaly from 2001 to 2022
	VPD_{anom_gs} (kPa)	Growing season vapor pressure deficit anomaly from 2001 to 2022
	$R_{a\_anom\_gs}$ (MJ m^{-2})	Growing season surface net solar radiation anomaly from 2001 to 2022

Supplementary Table 2. Parameters of SMA and classical gap fraction for fractional vegetation cover calculation for different grassland types across China. $G(\theta, \theta_1)$ refers to the orthogonal projection of a unit leaf area in the direction of θ.

**Grassland types**	**$NDVI_{soil}$**	**$NDVI_{veg}$**	**G (0, θ_1)**
Temperate meadow-steppe	0.02	0.895	1.00
Temperate steppe	0.01	0.88	0.42
Temperate desert-steppe	0.145	0.515	1.00
Alpine steppe	0.01	0.5	1.00
Alpine desert-steppe	0.01	0.5	0.63
Lowland meadow	0.13	0.875	0.67
Temperate montane meadow	0.01	0.83	1.00
Alpine meadow	0.01	0.715	1.00

Supplementary Table 3. Effective coefficients of change rates (Δ) in canopy height and fractional vegetation cover, resulting from environmental factors and grazing pressure, independently and interactively, across areas with increased and decreased canopy height from 2001 to 2022. The relationships among environmental factors, grazing pressure, canopy height, and fractional vegetation cover were calculated using linear mixed effects models (LMMs), where the environmental factors and grazing pressure were normalized using the z-score method by subtracting the mean of each term and dividing by the standard deviation for cross-comparison of the independent and interactive effects. A higher absolute value of independent and interactive effects indicates a stronger effect on promoting or diminishing canopy height and fractional vegetation cover changes. The independent and interactive effects are asterisked (*) if statistically significant ($P < 0.05$). The positive (+) and negative signs (-) indicate the direction of the corresponding trend.

Driving factors	Areas with increased canopy height		Areas with decreased canopy height	
	Effects on Δ canopy height (+)	Effects on Δ fractional vegetation cover (+)	Effects on Δ canopy height (-)	Effects on Δ fractional vegetation cover (+)
ΔT_a	-0.38*	-0.19	-0.59*	-0.45*
ΔG_p	-0.19	-0.16	-0.43*	-0.37*
ΔP_r	0.50*	0.27*	0.18	0.20*
ΔR_a	0.58*	0.18	0.66*	0.24*
ΔC_a	0.26*	0.20*	0.29*	0.27*
$\Delta T_a \times \Delta G_p$	-0.35*	-0.14	-0.67*	-0.29*
$\Delta T_a \times \Delta P_r$	-0.26*	-0.20*	-0.06	-0.04
$\Delta T_a \times \Delta R_a$	-0.03	-0.13	-0.05	-0.01
$\Delta T_a \times \Delta C_a$	-0.90*	-0.21*	-0.77*	-0.70*
$\Delta G_p \times \Delta P_r$	0.04	-0.09	0.06	0.26*
$\Delta G_p \times \Delta R_a$	-0.09	-0.06	-0.18	-0.41*
$\Delta G_p \times \Delta C_a$	0.09	-0.26*	-0.17	-0.29*
$\Delta P_r \times \Delta R_a$	0.20	0.15	0.03	-0.02
$\Delta P_r \times \Delta C_a$	0.03	0.15	0.38*	-0.33*
$\Delta R_a \times \Delta C_a$	0.30*	0.15	0.56*	0.13
