# Supplementary material for: Declining grassland canopy height in China under asymmetric biomass allocation
Source: Nat Commun. 2026 Mar 3;17:3364. doi: 10.1038/s41467-026-70275-9 (PMC13066366; doi:10.1038/s41467-026-70275-9)
Supplement: Supplementary file 3 — Reporting Summary [file 41467_2026_70275_MOESM3_ESM.pdf]

nature portfolio

Corresponding author(s): Fei Li; Yingjun Zhang

Last updated by author(s): Feb 3, 2026

Reporting Summary

Nature Portfolio wishes to improve the reproducibility of the work that we publish. This form provides structure for consistency and transparency in reporting. For further information on Nature Portfolio policies, see our Editorial Policies and the Editorial Policy Checklist.

Statistics

For all statistical analyses, confirm that the following items are present in the figure legend, table legend, main text, or Methods section.

n/a	Confirmed	
[ ]	[x]	The exact sample size (*n*) for each experimental group/condition, given as a discrete number and unit of measurement
[ ]	[x]	A statement on whether measurements were taken from distinct samples or whether the same sample was measured repeatedly
[ ]	[x]	The statistical test(s) used AND whether they are one- or two-sided<br>*Only common tests should be described solely by name; describe more complex techniques in the Methods section.*
[x]	[ ]	A description of all covariates tested
[ ]	[x]	A description of any assumptions or corrections, such as tests of normality and adjustment for multiple comparisons
[ ]	[x]	A full description of the statistical parameters including central tendency (e.g. means) or other basic estimates (e.g. regression coefficient) AND variation (e.g. standard deviation) or associated estimates of uncertainty (e.g. confidence intervals)
[ ]	[x]	For null hypothesis testing, the test statistic (e.g. *F*, *t*, *r*) with confidence intervals, effect sizes, degrees of freedom and *P* value noted<br>*Give P values as exact values whenever suitable.*
[x]	[ ]	For Bayesian analysis, information on the choice of priors and Markov chain Monte Carlo settings
[x]	[ ]	For hierarchical and complex designs, identification of the appropriate level for tests and full reporting of outcomes
[ ]	[x]	Estimates of effect sizes (e.g. Cohen's *d*, Pearson's *r*), indicating how they were calculated

Our web collection on statistics for biologists contains articles on many of the points above.

Software and code

Policy information about availability of computer code

Data collection	Data were compiled and managed in Python 3.10.16 and R v4.5.1. Remote-sensing data handling and extraction were performed on Google Earth Engine (GEE) using JavaScript.
Data analysis	Machine-learning analyses were performed in Python 3.10.16, using RandomForestRegressor from scikit-learn v1.6.1 and RandomOverSampler from imbalanced-learn v0.13.0. Fractional vegetation cover calculations were implemented on Google Earth Engine (GEE) using JavaScript, applying the normalizedDifference function. Statistical analyses were conducted in R v4.5.1, with structural equation models implemented using psem in the piecewiseSEM package and linear mixed-effects models fitted using lme in the nlme package. The codes are available at https://doi.org/10.5281/zenodo.18453938.

For manuscripts utilizing custom algorithms or software that are central to the research but not yet described in published literature, software must be made available to editors and reviewers. We strongly encourage code deposition in a community repository (e.g. GitHub). See the Nature Portfolio guidelines for submitting code & software for further information.

Data

Policy information about availability of data

All manuscripts must include a data availability statement. This statement should provide the following information, where applicable:

- Accession codes, unique identifiers, or web links for publicly available datasets
- A description of any restrictions on data availability
- For clinical datasets or third party data, please ensure that the statement adheres to our policy

The MOD09A1 data can be accessed at https://doi.org/10.5067/MODIS/MOD09A1.061 and the MOD15A2H data from https://doi.org/10.5067/MODIS/MOD15A2H.061. GEDI Level 2A and Level 2B products are available at https://doi.org/10.5067/GEDI/GEDI02_A.002 and https://doi.org/10.5067/GEDI/GEDI02_B.002, respectively. The ERA5-Land dataset can be downloaded at https://cds.climate.copernicus.eu/. The CO2 data can be accessed at https://doi.org/10.24381/a90c7e33. The field measurements of aboveground biomass, fractional vegetation cover, canopy height and species richness are collected from the National Inventory of Grassland Resources by the National Forestry and Grassland Administration of China. The grazing intensity data is available at https://doi.org/10.6084/m9.figshare.26195684. The data for continuous interannual measurements of aboveground biomass and fractional vegetation cover is primarily obtained from the China Ecosystem Research Network (www.nesdc.org.cn). The gridded dataset of grassland canopy height across China (2001–2022) generated in this study is available at https://doi.org/10.5281/zenodo.18454934.

Research involving human participants, their data, or biological material

Policy information about studies with human participants or human data. See also policy information about sex, gender (identity/presentation), and sexual orientation and race, ethnicity and racism.

Reporting on sex and gender	Not applied.
Reporting on race, ethnicity, or other socially relevant groupings	Not applied.
Population characteristics	Not applied.
Recruitment	Not applied.
Ethics oversight	Not applied.

Note that full information on the approval of the study protocol must also be provided in the manuscript.

Field-specific reporting

Please select the one below that is the best fit for your research. If you are not sure, read the appropriate sections before making your selection.

☐ Life sciences ☐ Behavioural & social sciences ☒ Ecological, evolutionary & environmental sciences

For a reference copy of the document with all sections, see nature.com/documents/nr-reporting-summary-flat.pdf

Ecological, evolutionary & environmental sciences study design

All studies must disclose on these points even when the disclosure is negative.

Study description	This study follows an observational, hierarchical sampling design. Grassland sites were distributed nationwide across major grassland types. Within each site, plots were established at approximately 500 m intervals, and each plot contained three sampling points separated by at least 10 m. Individual 1 m × 1 m quadrats served as the basic sampling units. A total of 24,125 sampling units provided extensive spatial replication across grassland types, locations, and years (2009–2016).
Research sample	A total of 24,125 field plots were surveyed.
Sampling strategy	The grassland samples were collected through a randomized, nationwide, full-coverage survey design. Spatially, sampling locations were selected following a random rule, with plots established at approximately 500 m intervals. At each plot, three sampling points were randomly selected, and individual samples within a plot were separated by at least 10 m to ensure spatial independence and representativeness. The final dataset comprises 24,125 grassland sampling points, evenly distributed across major grassland types nationwide. No a priori statistical power analysis was conducted, as the sample size was determined by the standardized design of the national grassland inventory. The large sample size, extensive spatial coverage, and balanced representation of grassland types provide sufficient statistical power to support robust spatial analyses and minimize sampling uncertainty, consistent with previous large-scale ecological and grassland monitoring studies.
Data collection	Data were collected by trained personnel involved in the national grassland survey in China, following standardized field protocols. Aboveground biomass was measured using 1 m × 1 m quadrats. All aboveground vegetation within each quadrat was clipped at ground level using scissors, transported to the laboratory, oven-dried at 85 °C to constant weight, and weighed using an electronic balance. The biomass measurements were recorded manually on standardized field sheets. Canopy height was measured in situ at the center of each quadrat using a ruler, and the values were recorded immediately in the field. Fractional vegetation cover was estimated using the point quadrat method, in which a grid frame with vertically arranged pins was placed over the plot, and the ratio

	of pin hits on vegetation to the total number of pins was recorded. All cover measurements were documented on paper records at the time of observation. All field records were subsequently digitized and uploaded to a centralized electronic management system of the national grassland survey program. The final dataset was quality-controlled, compiled, and standardized by the national authority to produce a consistent, nationwide grassland inventory.
Timing and spatial scale	All empirical data were collected between 2009 and 2016. Sampling was conducted annually during the peak growing season, with each annual survey carried out from 1 July to 31 August. No gaps occurred between sampling periods, and all years followed the same sampling schedule and protocols. Data were collected at the plot level using 1 m × 1 m quadrats and were spatially distributed across grassland sites nationwide. The survey achieved uniform national coverage across all major grassland types, providing a consistent, country-scale dataset.
Data exclusions	No data were excluded from analysis.
Reproducibility	This study is based on standardized, large-scale field survey data rather than laboratory experiments. Reproducibility was ensured through the use of uniform sampling designs, consistent field protocols, and standardized measurement procedures applied across all sites, years, and survey teams. Measurements were repeated across multiple plots, grassland types, and years (2009–2016) following identical protocols. All repeated field surveys were conducted successfully, and no failed attempts at data collection or measurement were recorded. Data consistency was further ensured through centralized quality control and compilation by the national grassland survey authority.
Randomization	Sampling locations were selected following a randomized survey design to ensure representative spatial coverage across grassland types. Extensive spatial and temporal replication was used to capture environmental variability.
Blinding	Blinding was not relevant to this study, as all data were derived from objective remote sensing sources and standardized field measurements, with no subjective assessment involved. In addition, the selection of plot locations was entirely based on the principle of random and uniform distribution, eliminating any potential subjective influence.

Did the study involve field work? ☐ Yes ☒ No

Reporting for specific materials, systems and methods

We require information from authors about some types of materials, experimental systems and methods used in many studies. Here, indicate whether each material, system or method listed is relevant to your study. If you are not sure if a list item applies to your research, read the appropriate section before selecting a response.

Materials & experimental systems

n/a	Involved in the study
☒	☐ Antibodies
☒	☐ Eukaryotic cell lines
☒	☐ Palaeontology and archaeology
☒	☐ Animals and other organisms
☒	☐ Clinical data
☒	☐ Dual use research of concern
☒	☐ Plants

Methods

n/a	Involved in the study
☒	☐ ChIP-seq
☒	☐ Flow cytometry
☒	☐ MRI-based neuroimaging

Plants

Seed stocks	Not applicable. No seed stocks or cultivated plant materials were used; all data were obtained from natural grasslands.
Novel plant genotypes	This study did not involve the development or use of any novel plant genotypes.
Authentication	Not applicable. This study did not involve the use of seed stocks, genetic modifications, or novel genotypes requiring authentication.
